# Superelectrophilic carbocations: preparation and reactions of a substrate with six ionizable groups

**DOI:** 10.3762/bjoc.15.153

**Published:** 2019-07-09

**Authors:** Sean H Kennedy, Makafui Gasonoo, Douglas A Klumpp

**Affiliations:** 1 Department of Chemistry and Biochemistry, Northern Illinois University, DeKalb, IL 60178, USA

**Keywords:** cation, Friedel–Crafts, heterocycle, superacid, superelectrophile

## Abstract

A substrate has been prepared having two triarylmethanol centers and four pyridine-type substituent groups. Upon ionization in the Brønsted superacid CF_3_SO_3_H, the substrate undergoes two types of reactions. In the presence of only the superacid, the highly ionized intermediate(s) provide a double cyclization product having two pyrido[1,2-*a*]indole rings. With added benzene, an arylation product is obtained. A mechanism is proposed involving tetra-, penta-, or hexacationic species.

## Introduction

**Scheme 1:**
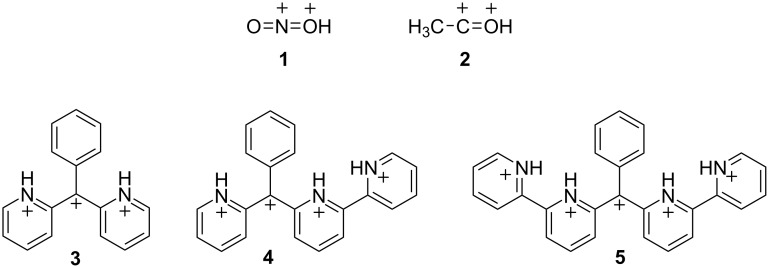
Superelectrophilic species.

During the 1970s and 80s, Olah and co-workers described the novel chemistry of highly-charged organic cationic species. This work lead to the concept of superelectrophilic reactivity [1–5]. Examples of superelectrophiles include the nitronium dication (**1**) and the acetylium dication (**2**, Scheme 1). Both of these species have been proposed as superelectrophilic intermediates in the reactions of nitronium (NO_2_^+^) and acetylium (CH_3_CO^+^) salts in superacids. In sufficiently acidic media, cationic electrophiles such as the nitronium ion may undergo protonation, leading to the nitronium dication (**1**), and a greatly enhanced electrophilic reactivity. In superacidic solutions, nitronium salts have been shown to react with deactivated arenes and saturated hydrocarbons (including methane) [6–9].

Numerous studies from our group and others have shown that relatively stable cationic centers – such as ammonium, phosphonium, and pyridinium groups – may also be part of superelectrophilic systems [10]. Recently, we described the chemistry of tri-, tetra-, and pentacationic electrophiles based on the triarylmethyl cation scaffold (**3–5**, Scheme 1) [11–12]. These systems utilized pyridyl rings to produce increasing amounts of positive charge adjacent to the carbocation center. Both theoretical calculations and experimental results indicated that the carbocation center undergoes a high degree of delocalization into the neighboring phenyl group. Both the trication **3** and the tetracation **4** were directly observed from FSO_3_H–SbF_5_ solution using low temperature NMR. Experimental observations also revealed an exceptionally high acidity of the pyridinium N–H bonds. Here, we describe the preparation and chemistry of a substrate with six ionizable groups – four pyridyl rings and two carbinol centers.

## Results and Discussion

**Scheme 2:**
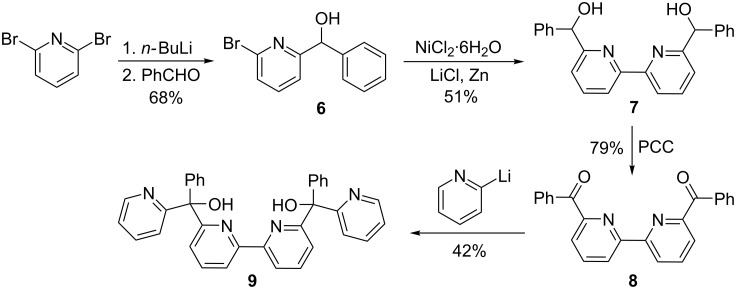
Synthesis of diol substrate **9**.

The desired substrate was prepared in four steps from 2,6-dibromopyridine (Scheme 2). Utilizing 2-lithio-6-bromopyridne, product **6** is formed in modest yield by reaction with benzaldehyde. A nickel-catalyzed procedure gives the dipyridyl intermediate **7** [13]. This is easily oxidized to the diketone **8** and reaction of this substance with 2-lithiopyridine gives the precursor **9**. The diol **9** is a substrate with six ionizable groups.

**Scheme 3:**
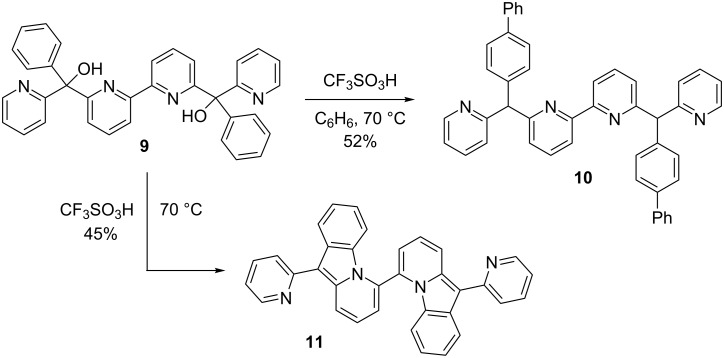
Isolated yields of products from diol **9**.

Upon ionization in superacidic CF_3_SO_3_H (triflic acid), compound **9** undergoes two types of reactions. When the substrate is ionized in the presence of benzene and CF_3_SO_3_H, the arylation product **10** is formed as the major product (Scheme 3). Presumably, compound **10** is formed as a mixture of *meso* and *dl* stereoisomers. Similar reaction products were observed in our studies of tri-, tetra-, and pentacationic systems [11–12]. This product, **10**, is the result of charge migration involving the carbocation center and the phenyl group (vide infra). When compound **9** is treated with only superacid, the bis(pyrido[1,2-*a*]indole) **11** is formed as the major product. Likewise, the tri-, tetra-, and pentacationic systems **3**–**5** provide the pyrido[1,2-*a*]indole ring system. Interestingly, there was no evidence of the cyclization product **11** (NMR analysis) when compound **9** reacts with superacid in the presence of benzene – product **10** is formed as the major product.

**Scheme 4:**
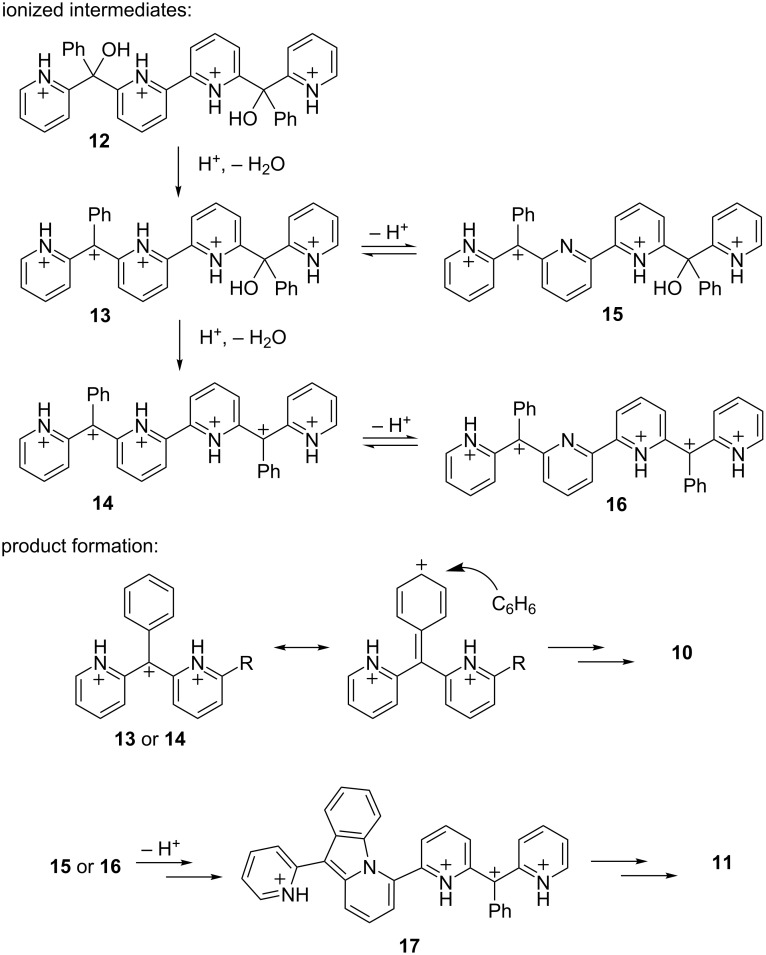
Proposed mechanisms leading to products **10** and **11**.

These products may be explained by mechanisms involving highly ionized intermediates. It is proposed that compound **9** initially reacts in excess superacid to give the tetracationic species **12** (Scheme 4). Protonation of the hydroxy groups leads to immediate ionization and formation of the carbocation centers. For related conversions, computational and experimental data indicated that the protonated hydroxy groups (oxonium ions) are not persistent intermediates, but rather cleavage of the carbon–oxygen bond is almost instantaneous [12]. It is assumed that ionization to the carbocations occurs in a stepwise process, first providing pentacation **13** then the hexacation **14**. The product forming steps occur through either **13** or **14**. For the arylation product **10**, charge delocalization at the carbocation leads to nucleophilic attack at the *para*-position of the phenyl group. This S_E_Ar step is followed by protonation at the methine position and deprotonation of the *para*-carbon to complete the arylation step. For the cyclization product **11**, theoretical calculations indicate that cyclization requires deprotonation at the pyridinium ring [11]. Thus, either the tetracation **15** or the pentacation **16** is the likely precursor to the pyrido[*1,2-a*]indole ring system. The conversion to product **11** also involves a stepwise process – initially forming compound **17** then a second cyclization gives the final product **11**.

**Scheme 5:**
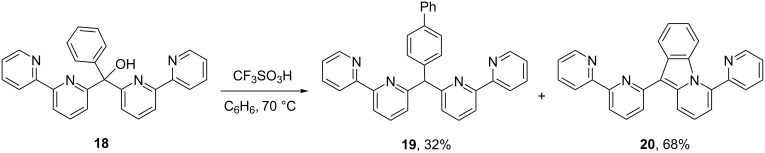
Products and relative yields from the reaction of alcohol **18** with CF_3_SO_3_H and C_6_H_6_ [12].

As noted above, no cyclization product **11** was observed when the chemistry was done in the presence of benzene. Only product **10** was obtained. This observation is in stark contrast to our earlier results involving both the tetracation **4** and pentacation **5**. When these highly charged ions are generated in CF_3_SO_3_H and C_6_H_6_, significant quantities of the cyclization products are formed with the respective arylation products. For example, alcohol **18** provides a mixture of products **19** and **20** in a 32:68 ratio, presumably through the pentacation **5** (Scheme 5). Even with the use of the stronger Brønsted acid, CF_3_SO_3_H–SbF_5_, significant quantities of the cyclization product **20** are observed. This raises an obvious question: why does substrate **9** provide exclusively the arylated product **10** in the presence of benzene, while substrate **18** leads to a significant proportion of cyclization product **20**?

**Scheme 6:**
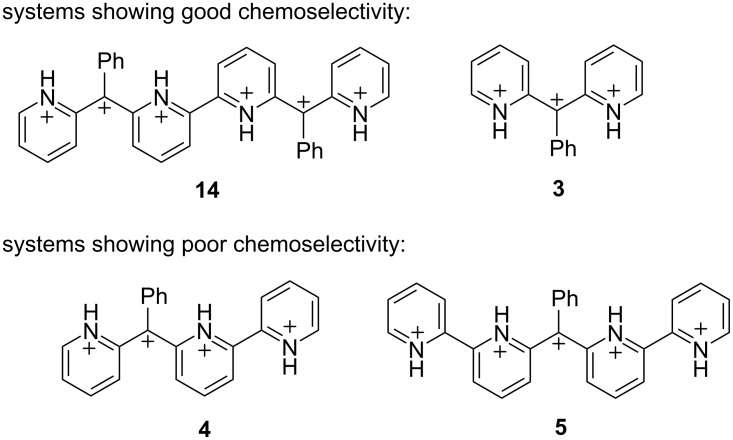
Comparison of superelectrophilic carbocations (**3–5** and **14**) and their chemistry.

Our proposed mechanism of cyclization involves deprotonation of an N–H bond at the pyridinium ring. Although pyridinium deprotonation is not generally expected in a Brønsted superacid, it can occur in these systems because of the large amount of cationic charge on these structures. In comparing the chemistry of compounds **9** and **18**, compound **9** can lead to the hexacationic intermediate **14** while compound **18** can lead to the pentacationic intermediate **5**. Examination of these two structures suggests that the phenyl groups have a profound effect on stabilizing the ions. Thus, the hexacationic system **14** is stabilized by two phenyl groups, while the pentacationic system **5** has one stabilizing phenyl group (Scheme 6). The ratio of charges is 2:1 (pyridinium/benzylic carbocation) for the hexacation **14**, while the ratio of charges is 4:1 (pyridinium/benzylic carbocation) for the pentacation **5**. Similarly, we previously reported good chemoselectivity for trication **3** – no cyclization product observed in the presence of benzene – but poor chemoselectivity for the tetracation **4**. The ratio of charges in these systems are consistent: a charge ratio of 2:1 shows good chemoselectivity (trication **3**), while a charge ratio of 3:1 shows poor chemoselectivity (tetracation **4**). As we previously reported, a reaction of tetracation **4** with benzene in CF_3_SO_3_H leads to a mixture of arylated product (52%) and cyclization product (48%). Thus, we observed clean arylation chemistry when the carbocation sites are flanked by not more than two pyridinium groups. This also means that increasing the number of adjacent pyridinium groups destabilizes the system as a whole and leads to greater N–H deprotonation. Tetracation **4** and pentacation **5** tend to undergo N–H deprotonation more readily, and consequently, this leads to rapid cyclization reactions.

**Scheme 7:**
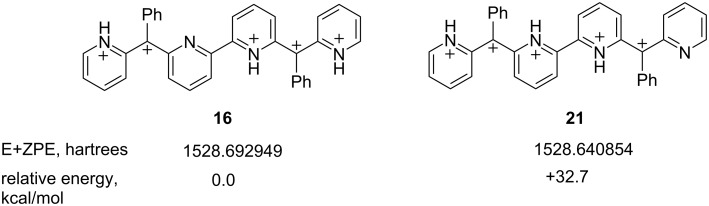
DFT calculated relative energies of pentacations **16** and **21** [14].

Regarding the site of deprotonation, hexacation **14** could potentially undergo N–H deprotonation at the inside pyridinium ring (**16**) or the outside pyridinium ring (**21**, Scheme 7). While inside deprotonation should give the observed cyclization product **11**, outside deprotonation would give an entirely different product, one having the pyrido[1,2-*a*]indole ring at the end of the structure. Compound **11** is the only major product observed from the superacid-promoted reaction of diol **9**. This suggests outside deprotonation – and formation of ion **21** – does not occur.

The preference for inside deprotonation may be understood to be a consequence of charge–charge repulsive effects. In the case of **21**, the five cationic charges are on neighboring positions, while in the case of **16**, the five cationic charges are separated into groups of three and two charges. The increased stability of the separated cationic charge is evident in the DFT calculated energies of the ions. At the B3LYP 6-311G (d,p) level, ion **16** is calculated to be 32.7 kcal·mol^−1^ more stable than ion **21** [8]. Thus, highly charged organic ions may benefit by having groups of charges separated into smaller clusters rather than having all of the charges grouped together.

## Conclusion

We have prepared a substrate with six ionizable sites. Reaction of the substrate in superacidic CF_3_SO_3_H leads to cyclization or arylation products, depending on the presence or absence of benzene. A mechanism is proposed involving tetra-, penta-, and hexacationic reactive intermediates. Most notably, this system shows remarkably good chemoselectivity in its reaction with benzene (only arylation product is observed). This is attributed to the presence of two carbocationic sites stabilized by benzylic-type resonance. Thus, molecular structures having a very large overall charge may be viable if stabilizing groups are incorporated into the structure.

## Experimental

**General.** All reactions were performed using oven-dried glassware under an argon atmosphere. Trifluoromethanesulfonic acid (triflic acid) was freshly distilled prior to use. All commercially available compounds and solvents were used as received. ^1^H and ^13^C NMR were done using either 300 MHz or 500 MHz spectrometer; chemical shifts were made in reference to NMR solvent signals. Mass spectra were obtained from a commercial analytical laboratory. The synthesis of compound **9** is detailed in Supporting Information File 1.

**Preparation of 6,6'-bis([1,1'-biphenyl]-4-yl(pyridin-2-yl)methyl)-2,2'-bipyridine (10):** In a pressure tube at 25 °C, compound **9** (52.2 mg, 0.10 mmol) was dissolved in benzene (1 mL, 11.2 mmol), stirred for 5 min before triflic acid (1 mL, 11 mmol) was slowly added and the tube was then tightly closed. Following 24 h of stirring at 60–70 °C, the reaction was cooled to room temperature, poured on about 10 g of ice and then neutralized with 10 M NaOH solution. The resulting aqueous solution was then partitioned between chloroform and distilled water in a separatory funnel. The aqueous fraction was subjected to two further extractions after which the organic fractions were combined, washed with brine, dried over anhydrous sodium sulfate and filtered. The solvent was removed by rotary evaporation and the product purified by column chromatography (*R*_f_ 0.21, hexane/ethyl acetate 1:1). Compound **10** was isolated in 52% yield as oil. ^1^H NMR (300 MHz, CDCl_3_) δ 5.85 (s, 1H), 7.17–7.21 (m, 1H), 7.30–7.51 (m, 9H), 7.53–7.60 (m, 4H), 7.64–7.70 (m, 1H), 8.61 (d, *J* = 4.02 Hz, 1H); ^13^C NMR (75 MHz, CDCl_3_) δ 60.7, 121.9, 123.1, 124.3, 126.1, 127.1, 127.28, 127.33, 128.8, 129.6, 136.8, 138.8, 139.9, 140.2, 140.7, 141.4, 149.3, 161.1, 163.4; low-resolution ESIMS *m*/*z*: 643 [M + 1], 553, 477, 475, 401, 324, 323; high-resolution CIMS *m*/*z*: [M + 1] calcd for C_46_H_35_N_4_, 643.2862; found, 643.2856.

**Preparation of 10,10'-di(pyridin-2-yl)-6,6'-bipyrido[1,2-a]indole, (11):** In a pressure tube at 25 °C, compound **9** (200.1 mg, 0.481 mmol) was dissolved in triflic acid (1 mL, 11 mmol) and the tube was then tightly closed. Following 24 h of stirring at 60–70 °C, the reaction was cooled to room temperature, poured on about 10 g of ice and then neutralized with 10 M NaOH solution. The resulting aqueous solution was then partitioned between chloroform and distilled water in a separatory funnel. The aqueous fraction was subjected to two further extractions after which the organic fractions were combined, washed with brine, dried over anhydrous sodium sulfate and filtered. The solvent was removed by rotary evaporation and the product purified by column chromatography (*R*_f_ 0.30, hexane/ethyl acetate 1:1). The product **11** was isolated in 45% yield as brown oil. ^1^H NMR (300 MHz, CDCl_3_) δ 6.81–6.88 (m, 2H), 7.19 (t, *J* = 4.65 Hz, 1H), 7.35–7.40 (m, 1H), 7.51 (t, *J* = 7.50 Hz, 1H), 7.75– 7.82 (m, 2H), 8.24–8.29 (m, 2H), 8.81 (d, *J* = 2.55 Hz, 1H), 9.15 (d, *J* = 8.79 Hz, 1H); ^13^C NMR (75 MHz, CDCl_3_) δ 107.2, 116.0, 116.1, 116.7, 118.1, 119.2, 120.20, 120.28, 123.3, 123.6, 124.2, 128.3, 131.9, 136.4, 138.1, 149.7, 154.4; low-resolution ESIMS *m*/*z*: 487 [M + 1], 397, 353, 320, 319, 279, 244; high-resolution CIMS *m*/*z*: [M + 1], calcd for C_34_H_23_N_4_, 487.1923; found, 487.1917.

## Supporting Information

File 1 Experimental procedures, compounds characterization, and NMR spectra; computational methods and results.
