# Supplementary material for: Superelectrophilic carbocations: preparation and reactions of a substrate with six ionizable groups
Source: Beilstein J Org Chem. 2019 Jul 9;15:1515–20. doi: 10.3762/bjoc.15.153 (PMC6633208; doi:10.3762/bjoc.15.153)
Supplement: File 1 — Experimental procedures, compounds characterization, and NMR spectra; computational methods and results. [file Beilstein_J_Org_Chem-15-1515-s001.pdf]

BEILSTEIN JOURNAL OF ORGANIC CHEMISTRY

Supporting Information

for

Superelectrophilic carbocations: preparation and reactions of a substrate with six ionizable groups

Sean H. Kennedy, Makafui Gasonoo and Douglas A. Klumpp

Beilstein J. Org. Chem. **2019,** *15,* 1515–1520. doi:10.3762/bjoc.15.153

Experimental procedures, compounds characterization, and NMR spectra; computational methods and results

License and Terms: This is a supporting information file under the terms of the Creative Commons Attribution License (http://creativecommons.org/licenses/by/4.0). Please note that the reuse, redistribution and reproduction in particular requires that the authors and source are credited.
The license is subject to the *Beilstein Journal of Organic Chemistry* terms and conditions: (https://www.beilstein-journals.org/bjoc)

Page	
S1	Index
S2–S4	Experimental procedures and characterization data
S4	References
S5–S14	NMR spectra of new compounds
S15–S19	Computational methods and results

General. All reactions were performed using oven–dried glassware under an argon atmosphere. Trifluoromethanesulfonic acid (triflic acid) was freshly distilled prior to use. All commercially available compounds and solvents were used as received. ^{1}H and ^{13}C NMR were done using either 300 MHz or 500 MHz spectrometer; chemical shifts were made in reference to NMR solvent signals. Low–resolution mass spectra were obtained from a gas chromatography instrument equipped with a mass–selective detector, whereas high–resolution mass spectra were obtained from a commercial analytical laboratory (electron impact ionization; sector instrument analyzer type).

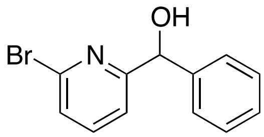

6

(6-bromopyridin-2-yl)(phenyl)methanol (6)

The title compound was prepared according to a literature procedure.[1]

To a 100 mL round-bottom flask under argon was charged 2,6-dibromopyridine (1.185 g, 5 mmol) and anhydrous diethyl ether (25 mL). This solution was cooled to −78 °C and stirred for 15 minutes. *n*-BuLi (1.6 M in hexanes, 3.1 mL, 5 mmol) was added dropwise over 1 min. The resulting brown mixture was stirred at −78 °C for 30 min, then benzaldehyde (0.55 mL, 5.5 mmol) was added dropwise. After 5 hours of stirring at room temperature, the blue-green reaction mixture was quenched with saturated NH_4Cl (3 mL) and extracted with ethyl acetate (25 mL). The combined organic layers were washed with brine and dried over Na_2SO_4. After filtration, the solvent was removed and the crude product was purified by column chromatography on silica gel to afford **6** (893 mg, 68%) as a yellow oil. R_f = 0.42 (80:20 hexanes/ethyl acetate). ^{1}H NMR (500 MHz, $CDCl_3$): δ 7.49 – 7.44 (m, 1H), 7.41 – 7.33 (m, 5H), 7.33 – 7.29 (m, 1H), 7.17 (d, *J* = 7.6 Hz, 1H), 5.75 (d, *J* = 4.1 Hz, 1H), 4.65 (d, *J* = 4.2 Hz, 1H). ^{13}C NMR (125 MHz, $CDCl_3$): δ 163.1, 142.2, 140.8, 139.2, 128.7, 128.1, 127.0, 126.8, 120.1, 75.1. Data was consistent with that reported in the literature.

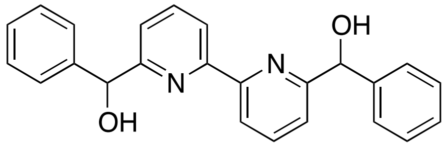

7

[2,2'-bipyridine]-6,6'-diylbis(phenylmethanol) (7)

The title compound was prepared according to a literature procedure.[2]

A 100 mL round-bottom flask was charged with $NiCl_2 \cdot 6H_20$ (39 mg, 0.163 mmol) and DMF (10 mL). The resulting solution was stirred and heated to 40 °C, and then **6** (0.86 g, 3.25 mmol), anhydrous LiCl (0.140 g, 3.25 mmol), and zinc dust (0.254 g, 3.9 mmol) were added. When the temperature rose to 50 °C, a grain of iodine crystal and two drops of acetic acid were added to the mixture. Within minutes, a color change to black occured. The mixture was stirred at 60 °C for 3 h until complete conversion of **6** was observed (monitored by TLC). To the cooled reaction mixture was added 1 M HCl aqueous solution (5 mL) to consume the remaining zinc dust. The resulting mixture was made basic with aqueous ammonia (25%) and extracted with DCM. The organic layers were collected, dried over anhydrous Na_2SO_4, and concentrated. The crude material was purified by flash chromatography to give **7** (0.612 g, 51%) as a solid. R_f = 0.18 (80:20 hexanes/ethyl acetate). ^{1}H NMR (300 MHz, $CDCl_3$): δ 8.59 (d, *J* = 4.7 Hz, 2H), 7.64 (ddd, *J* = 7.6, 7.5, 1.7 Hz, 2H), 7.42 – 7.33 (m, 10), 7.32 – 7.29 ((m, 2H), 7.24 – 7.21 (m, 2H), 7.18 (d, J = 7.7 Hz, 2H) 5.78 (s, 2H), 5.31 (br, s, 2H). ^{13}C NMR (75 MHz, $CDCl_3$): δ 160.8, 147.8, 143.2, 136.9, 128.6, 127.9, 127.1, 122.4, 121.4, 75.0. Data was consistent with that reported in the literature.

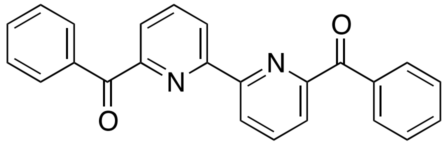

8

[2,2'-bipyridine]-6,6'-diylbis(phenylmethanone) (8)

In a round bottom flask, **7** (603 mg, 1.66 mmol) was dissolved in dichloromethane (15 mL). Celite (1.20 g) was added to the flask, followed by PCC (1.180 g, 3.3 equiv). After 18 hours the reaction mixture was concentrated to 2–3 mL of DCM using rotary evaporation. The residue was vacuum-filtered through a small silica plug, and washed three times with 50 mL ethyl acetate. The solvent was removed using rotary evaporation to afford **8** (477 mg, 79%, 1.31 mmol) as a pale yellow oil. R_f = (0.2, 90:10 ethyl acetate/methanol). ^{1}H NMR (300 MHz, $CDCl_3$): δ 8.73 (ddd, *J* = 4.8, 1.7, 0.9 Hz, 2H), 8.10 – 8.02 (m, 6H), 7.90 (td, *J* = 7.6, 7.6, 1.7 Hz, 2H), 7.63 – 7.56 (m, 2H), 7.52 – 7.45 (m, 6H). ^{13}C NMR (75 MHz, $CDCl_3$): δ 193.8, 155.1, 148.5, 137.0, 136.3, 132.9, 131.0, 128.1, 126.1, 124.6. Data was consistent with that reported in the literature.[3]

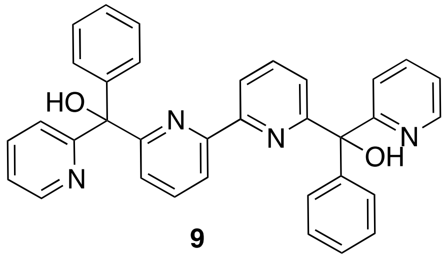

9

[2,2'-bipyridine]-6,6'-diylbis(phenyl(pyridin-2-yl)methanol) (9)

A solution of 2-bromopyridine (0.474 g, 3 mmol, 2.5 equiv) in diethyl ether (25 mL) was cooled to −78 °C. *n*-BuLi (1.6 M in hexanes, 1.9 mL, 2.5 equiv) was added dropwise over 1 minute and the reaction was stirred at to −78 °C for 30 minutes. A solution of **8** (441 mg, 1.21 mmol) in THF (10 mL) was added to the reaction over 10 minutes through a pressure-equalizing dropping funnel and the temperature was maintained at −78 °C for 30 minutes before slowly warming to room temperature and stirring for 18 hours. The reaction was quenched with addition of saturated NH_4Cl (5 mL). Ethyl acetate (20 mL) was added, the aqueous layer was separated and extracted once with ethyl acetate (15 mL), washed with brine, and dried over anhydrous sodium sulfate. The solvent was removed by rotary evaporation and the residue was purified with column chromatography to afford **9** (267 mg, 42%, 0.512 mmol) as a white solid; R_f = (0.23, 80:20 hexanes/ethyl acetate); mp 170-172 °C; ^1H NMR (500 MHz, $CDCl_3$): δ 8.58 – 8.55 (m, 2H), 8.20 (dd, *J* = 7.7, 0.8 Hz, 2H), 7.90 – 7.85 (m, 4H), 7.84 – 7.79 (m, 2H), 7.72 (m, 2H), 7.39 – 7.35 (m, 4H), 7.33 – 7.26 (m, 6H), 7.25 – 7.21 (m, 2H), 7.06 (s, 1H). ^{13}C NMR (125 MHz, $CDCl_3$): δ 163.1, 162.5, 153.3, 147.2, 146.2, 137.5, 136.3, 128.0, 127.9, 127.3, 123.4, 123.38, 123.1, 122.3, 119.4, 80.5. HRMS (ESI): m/z calculated for $C_{34}H_{26}N_4O_2$ (M+H) = 523.2134 ; found = 523.2069.

References:

1. *Organometallics*, **2016**, *35*(9), pp 1313-1322.
2. *J. Org. Chem.*, **2014**, *79* (2), pp 777–782.
3. *Angewandte Chemie, International Edition*, **2016**, *55*(3), 969-973.

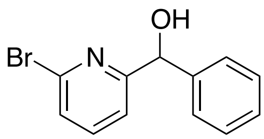
OH
Br
N

6

$CDCl_3$

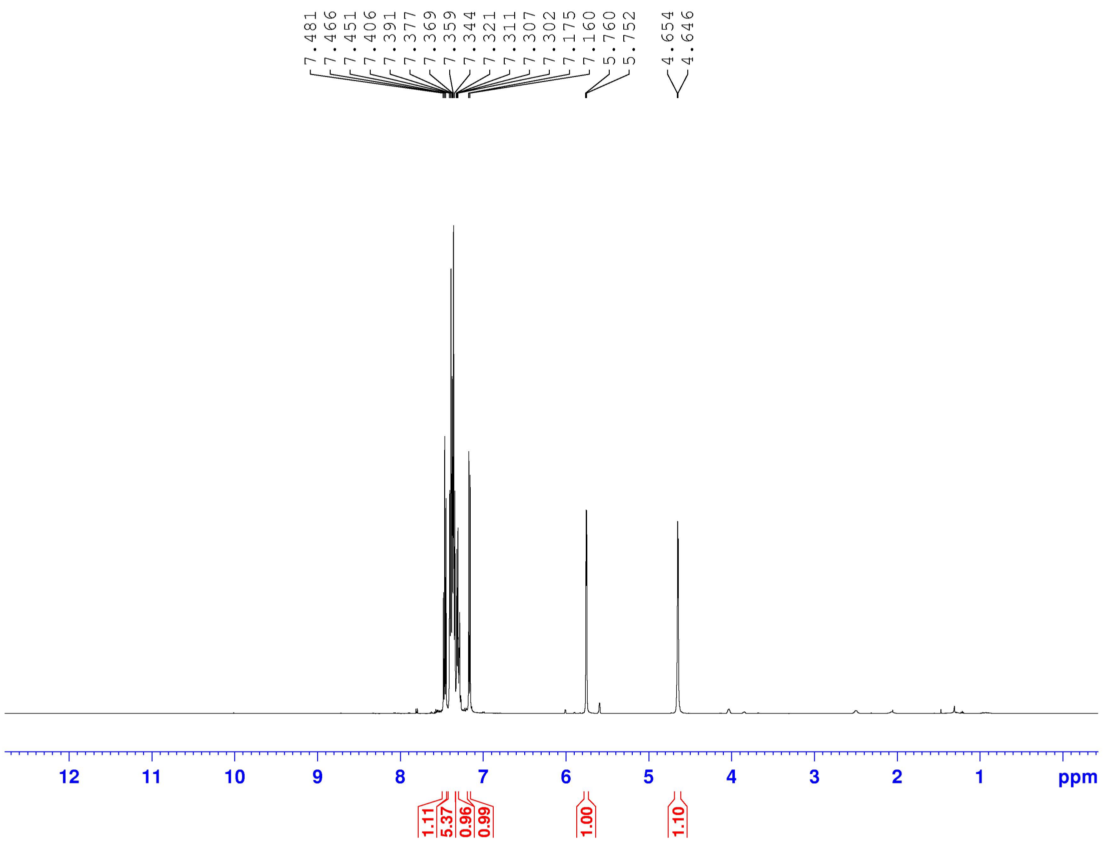
7.481
7.466
7.451
7.406
7.391
7.377
7.369
7.359
7.344
7.321
7.311
7.307
7.302
7.175
7.160
5.760
5.752
4.654
4.646
12
11
10
9
8
7
6
5
4
3
2
1
ppm
1.11
5.37
0.96
0.99
1.00
1.10

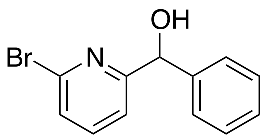
OH
Br
N

6

$CDCl_3$

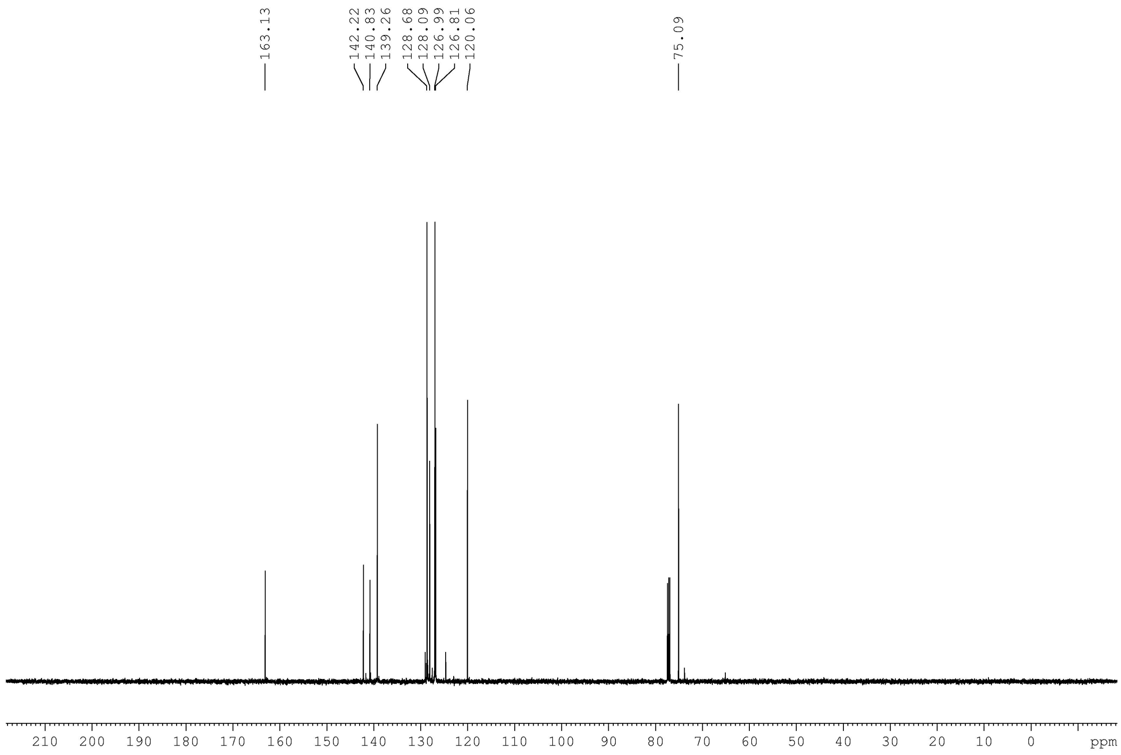
163.13
142.22
140.83
139.26
128.68
128.09
126.99
126.81
120.06
75.09
210 200 190 180 170 160 150 140 130 120 110 100 90 80 70 60 50 40 30 20 10 0 ppm

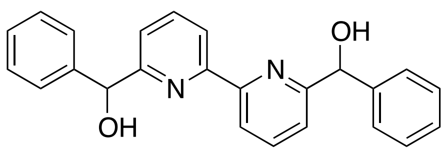

7

$CDCl_3$

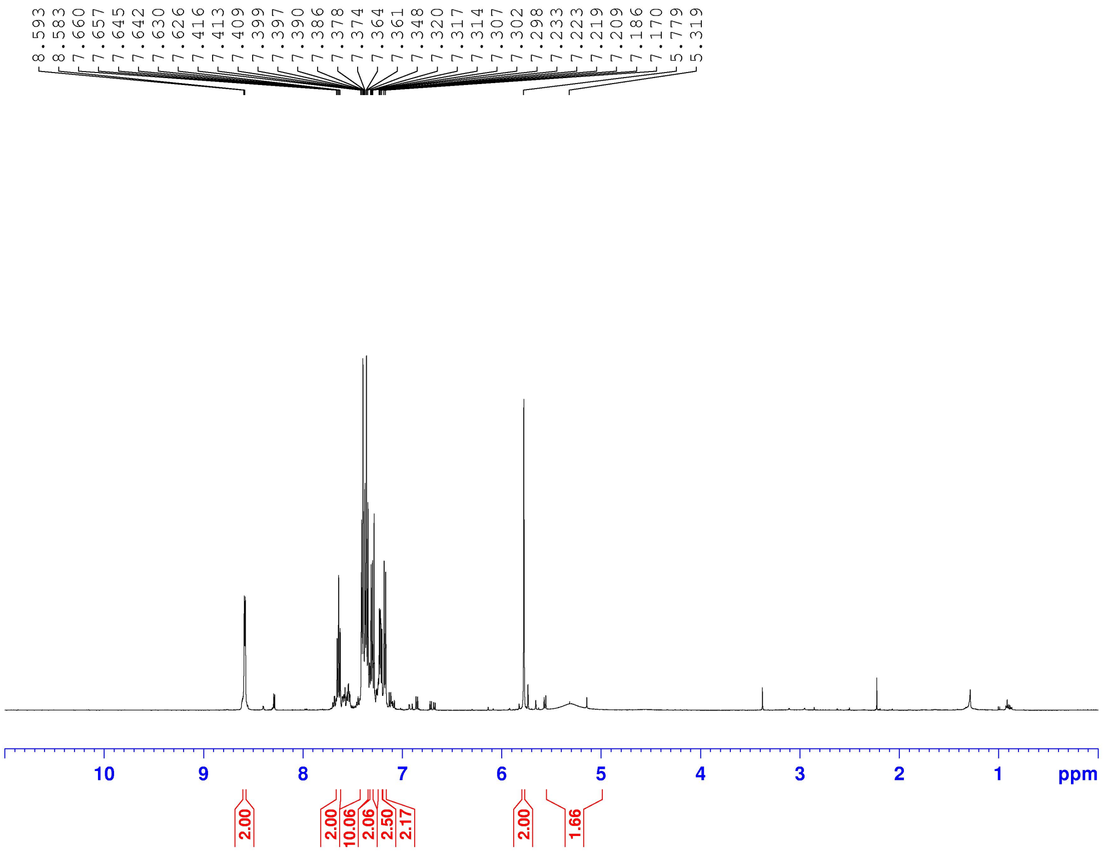
8.593
8.583
7.660
7.657
7.645
7.642
7.630
7.626
7.416
7.413
7.409
7.399
7.397
7.390
7.386
7.378
7.374
7.364
7.361
7.348
7.320
7.317
7.314
7.307
7.302
7.298
7.233
7.223
7.219
7.209
7.186
7.170
5.779
5.319
10
9
8
7
6
5
4
3
2
1
ppm
2.00
2.00
10.06
2.06
2.50
2.17
2.00
1.66

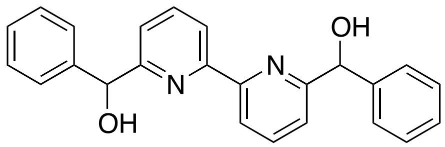

7

$CDCl_3$

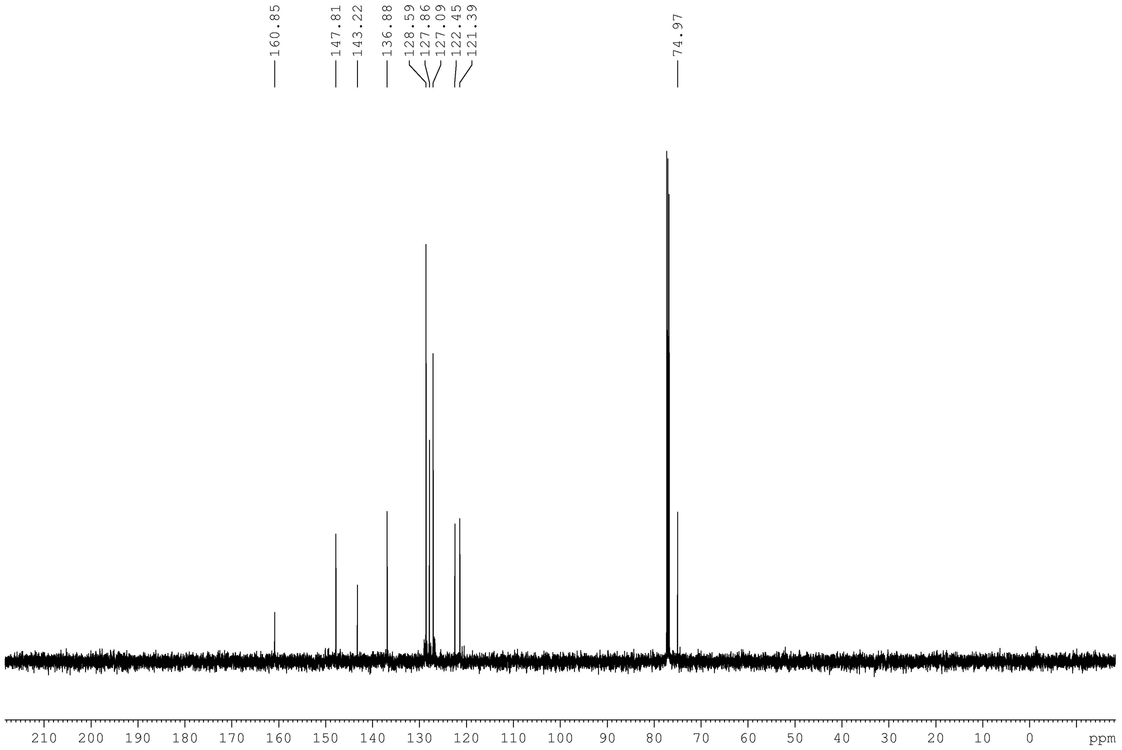

160.85
147.81
143.22
136.88
128.59
127.86
127.09
122.45
121.39
74.97
210 200 190 180 170 160 150 140 130 120 110 100 90 80 70 60 50 40 30 20 10 0 ppm

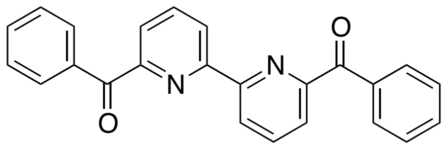
O
N
N
O

8

$CDCl_3$

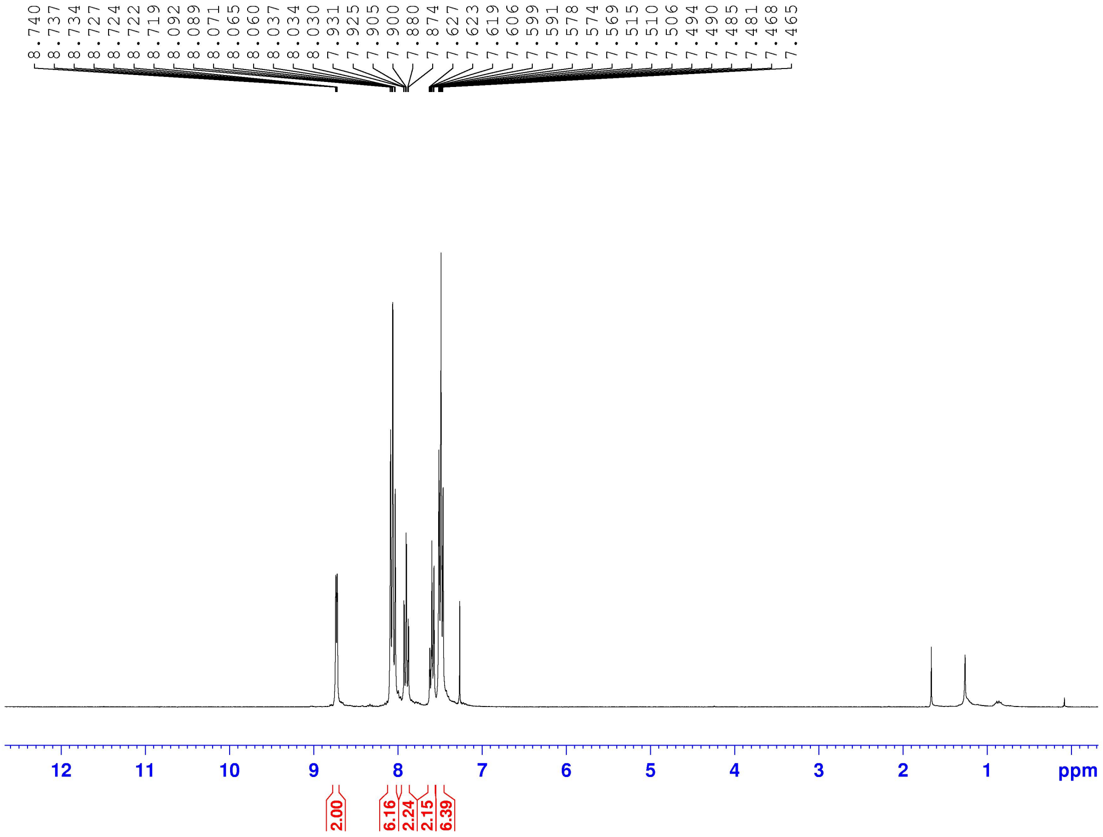
8.740
8.737
8.734
8.727
8.724
8.722
8.719
8.092
8.089
8.071
8.065
8.060
8.037
8.034
8.030
7.931
7.925
7.905
7.900
7.880
7.874
7.627
7.623
7.619
7.606
7.599
7.591
7.578
7.574
7.569
7.515
7.510
7.506
7.494
7.490
7.485
7.481
7.468
7.465
12 11 10 9 8 7 6 5 4 3 2 1 ppm
2.00
6.16
2.24
2.15
6.39

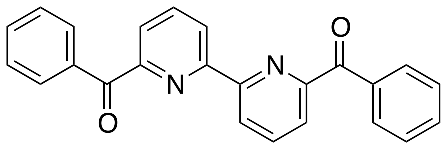

8

$CDCl_3$

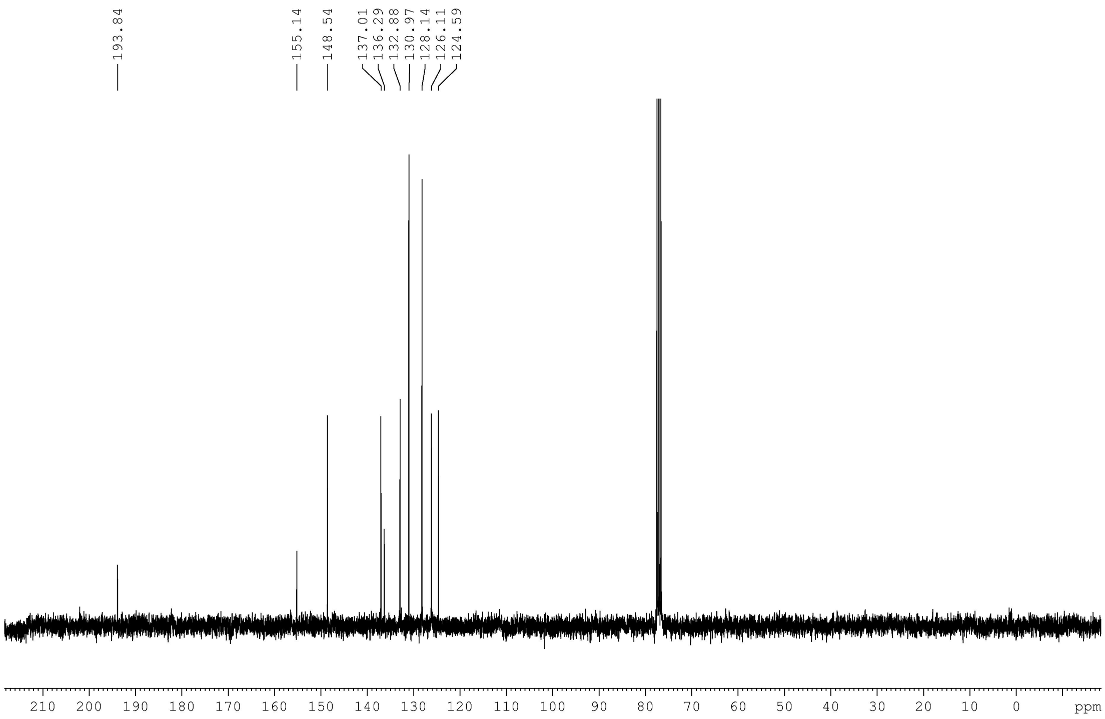
193.84
155.14
148.54
137.01
136.29
132.88
130.97
128.14
126.11
124.59
210 200 190 180 170 160 150 140 130 120 110 100 90 80 70 60 50 40 30 20 10 0 ppm

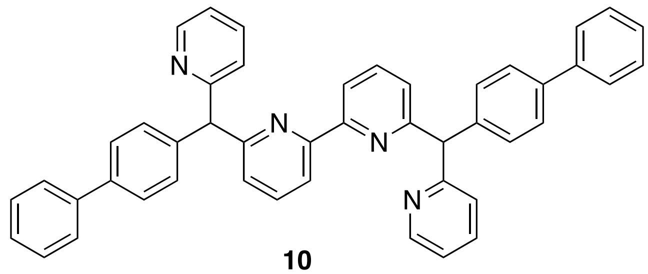

10

$CDCl_3$

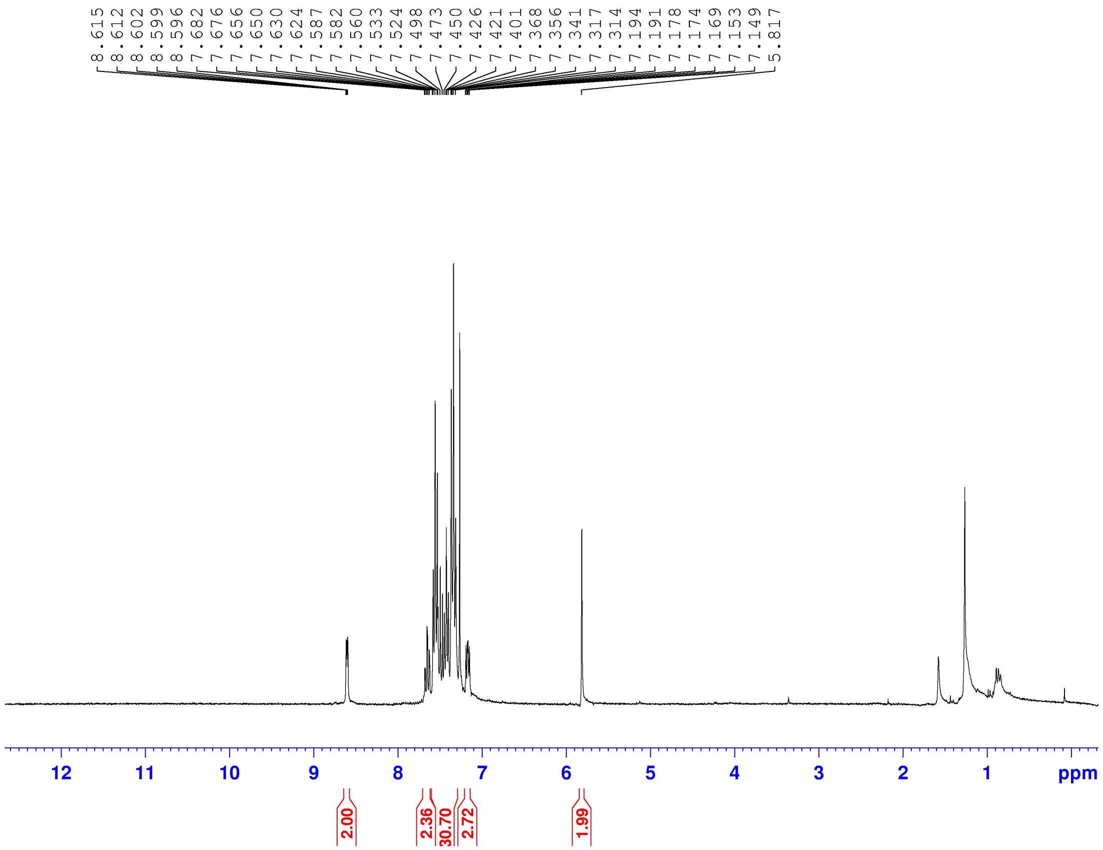
8.615
8.612
8.602
8.599
8.596
7.682
7.676
7.656
7.650
7.630
7.624
7.587
7.582
7.560
7.533
7.524
7.498
7.473
7.450
7.426
7.421
7.401
7.368
7.356
7.341
7.317
7.314
7.194
7.191
7.178
7.174
7.169
7.153
7.149
5.817
12
11
10
9
8
7
6
5
4
3
2
1
ppm
2.00
2.36
30.70
2.72
1.99

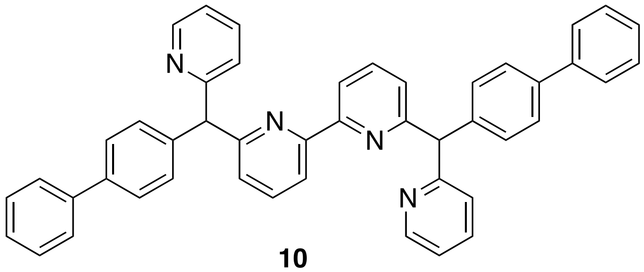

10

$CDCl_3$
(acetone impurity at 29 ppm)

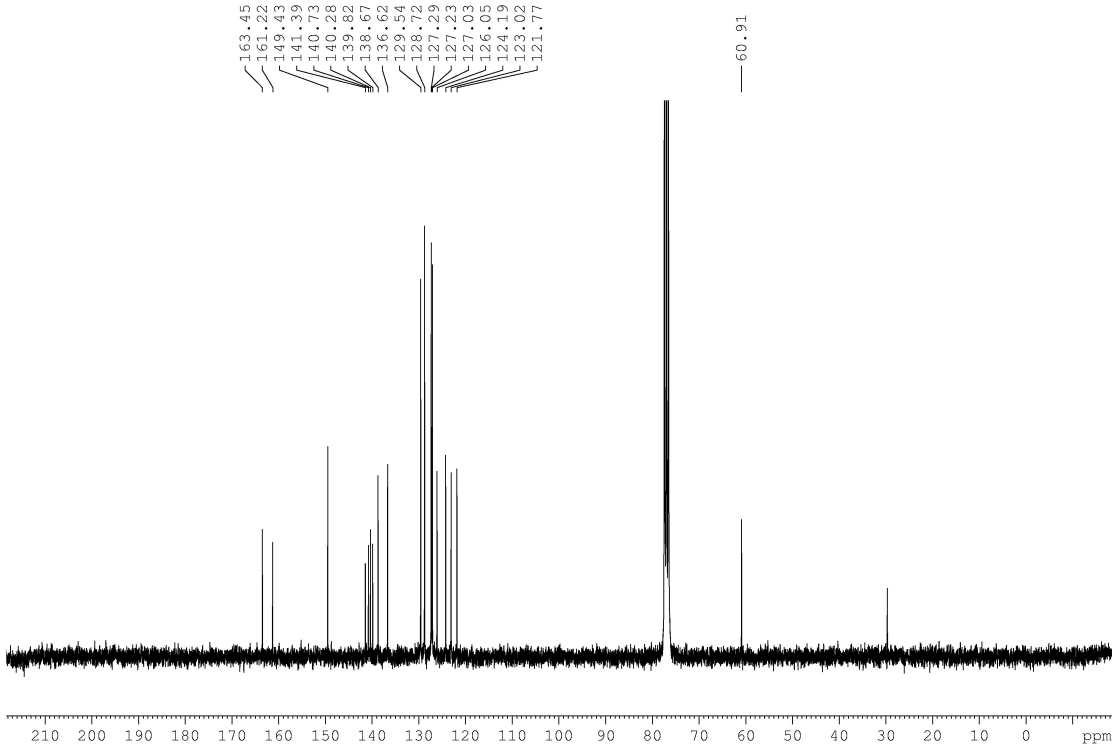

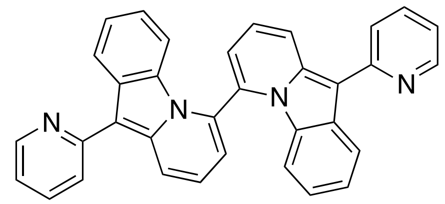

11

$CDCl_3$

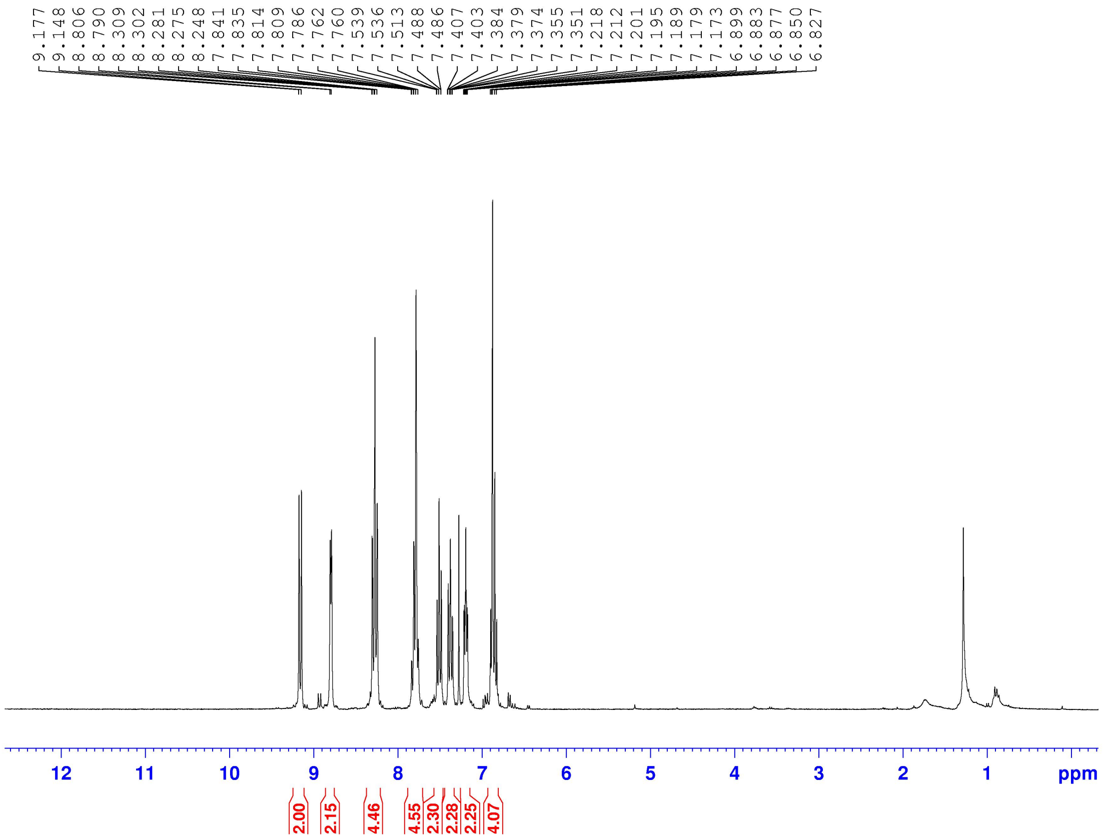
9.177
9.148
8.806
8.790
8.309
8.302
8.281
8.275
8.248
7.841
7.835
7.814
7.809
7.786
7.762
7.760
7.539
7.536
7.513
7.488
7.486
7.407
7.403
7.384
7.379
7.374
7.355
7.351
7.218
7.212
7.201
7.195
7.189
7.179
7.173
6.899
6.883
6.877
6.850
6.827
12 11 10 9 8 7 6 5 4 3 2 1 ppm
2.00
2.15
4.46
4.55
2.30
2.28
2.25
4.07

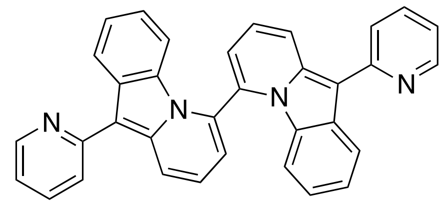

11

$CDCl_3$

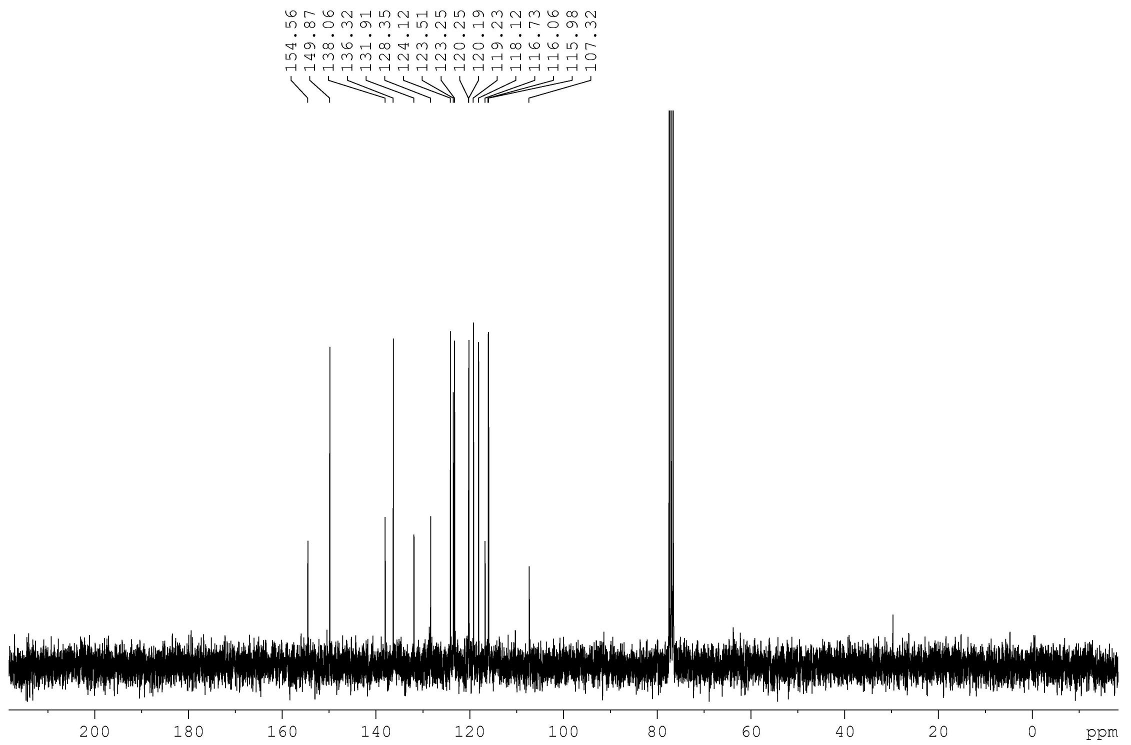
154.56
149.87
138.06
136.32
131.91
128.35
124.12
123.51
123.25
120.25
120.19
119.23
118.12
116.73
116.06
115.98
107.32
200
180
160
140
120
100
80
60
40
20
0
ppm

Computations

Computational Methods:

Calculations were done using the *Gaussian03W* program package.[4] Geometry optimizations were done using density function theory (DFT) and the hybrid functional B3LYP at the 6-311G** level.[5,6] Vibrational analyses were performed at the B3LYP/6-311G** level of theory. Zero-point-energy corrections are unscaled.

Literature Citations:

(4) Calculations done using Gaussian 03, Revision C.02, Frisch, M. J.; Trucks, G. W.; Schlegel, H. B.; Scuseria, G. E.; Robb, M. A.; Cheeseman, J. R.; Montgomery, Jr., J. A.; Vreven, T.; Kudin, K. N.; Burant, J. C.; Millam, J. M.; Iyengar, S. S.; Tomasi, J.; Barone, V.; Mennucci, B.; Cossi, M.; Scalmani, G.; Rega, N.; Petersson, G. A.; Nakatsuji, H.; Hada, M.; Ehara, M.; Toyota, K.; Fukuda, R.; Hasegawa, J.; Ishida, M.; Nakajima, T.; Honda, Y.; Kitao, O.; Nakai, H.; Klene, M.; Li, X.; Knox, J. E.; Hratchian, H. P.; Cross, J. B.; Bakken, V.; Adamo, C.; Jaramillo, J.; Gomperts, R.; Stratmann, R. E.; Yazyev, O.; Austin, A. J.; Cammi, R.; Pomelli, C.; Ochterski, J. W.; Ayala, P. Y.; Morokuma, K.; Voth, G. A.; Salvador, P.; Dannenberg, J. J.; Zakrzewski, V. G.; Dapprich, S.; Daniels, A. D.; Strain, M. C.; Farkas, O.; Malick, D. K.; Rabuck, A. D.; Raghavachari, K.; Foresman, J. B.; Ortiz, J. V.; Cui, Q.; Baboul, A. G.; Clifford, S.; Cioslowski, J.; Stefanov, B. B.; Liu, G.; Liashenko, A.; Piskorz, P.; Komaromi, I.; Martin, R. L.; Fox, D. J.; Keith, T.; Al-Laham, M. A.; Peng, C. Y.; Nanayakkara, A.; Challacombe, M.; Gill, P. M. W.; Johnson, B.; Chen, W.; Wong, M. W.; Gonzalez, C.; and Pople, J. A.; Gaussian, Inc., Wallingford CT, 2004.

(5) (a) Becke, A. D. *Phys. Rev. A* **1988**, *38*, 3098. (b) Becke, A. D. *J. Chem. Phys.* **1993**, *98*, 5648. (c) Lee, C.; Yang, W.; Parr, R. G. *Phys. Rev. B* **1988**, *37*, 785

(6) Raghavachari, K.; Binkley, J. S.; Seegar, R.; Pople, J. A. *J. Chem. Phys.* **1980**, *72*, 650.

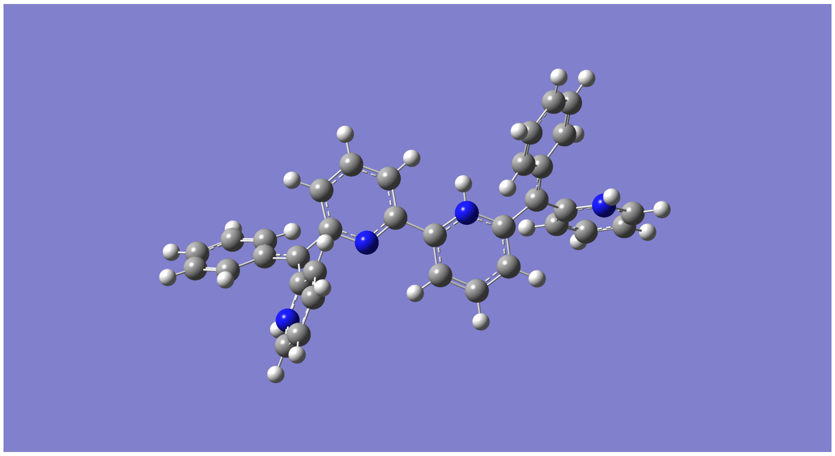

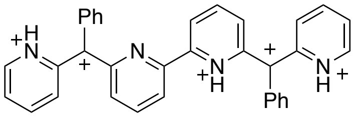

16

Imaginary Frequencies = 0

```
Zero-point correction=                           0.528037 (Hartree/Particle)
 Thermal correction to Energy=                    0.558172
 Thermal correction to Enthalpy=                  0.559116
 Thermal correction to Gibbs Free Energy=         0.463556
 Sum of electronic and zero-point Energies=          -1528.692949
 Sum of electronic and thermal Energies=             -1528.662814
 Sum of electronic and thermal Enthalpies=           -1528.661870
 Sum of electronic and thermal Free Energies=        -1528.757430
```

Standard orientation:

Center Number	Atomic Number	Atomic Type	Coordinates (Angstroms) X	Y	Z
1	6	0	4.318300	3.293108	-1.580721
2	6	0	3.902489	2.264814	-0.772811
3	6	0	4.631451	1.013227	-0.741699
4	6	0	5.768133	0.871299	-1.631753
5	6	0	6.141192	1.896819	-2.464971
6	6	0	5.434117	3.118883	-2.433328
7	1	0	3.816717	4.253624	-1.551010
8	1	0	3.095358	2.436488	-0.070632
9	1	0	6.277011	-0.082292	-1.708188
10	1	0	6.965852	1.770328	-3.157956
11	1	0	5.750938	3.937151	-3.074276
12	6	0	4.282528	-0.014141	0.154476
13	6	0	5.281824	-1.006237	0.611784
14	6	0	5.024069	-2.368287	0.748693
15	6	0	7.549994	-1.392401	1.292446
16	1	0	6.764485	0.420485	0.861836

17	6	0	6.048359	-3.253986	1.123412
18	1	0	4.039298	-2.756436	0.522133
19	6	0	7.320002	-2.766435	1.399137
20	1	0	8.503410	-0.929689	1.526815
21	1	0	5.842764	-4.316972	1.197817
22	1	0	8.130237	-3.419830	1.705226
23	6	0	2.919134	-0.150772	0.735787
24	6	0	2.713587	-0.427741	2.080212
25	6	0	1.406517	-0.490694	2.587804
26	1	0	3.558187	-0.555843	2.744882
27	6	0	0.518525	-0.085942	0.372355
28	1	0	1.967187	0.198964	-1.049180
29	6	0	0.322923	-0.327654	1.741501
30	1	0	1.251119	-0.680068	3.644756
31	1	0	-0.693819	-0.402458	2.100428
32	6	0	-0.632478	0.004018	-0.579390
33	6	0	-0.453212	0.035213	-1.972621
34	6	0	-1.571401	0.078331	-2.795719
35	1	0	0.524283	0.013057	-2.441316
36	6	0	-2.933438	0.000794	-0.806062
37	6	0	-2.833703	0.049364	-2.211161
38	1	0	-1.465528	0.119341	-3.873761
39	1	0	-3.720744	0.090441	-2.830935
40	6	0	-4.296766	-0.038080	-0.183994
41	6	0	-5.290409	-0.893407	-0.685004
42	6	0	-4.943593	-2.086235	-1.431138
43	6	0	-6.701520	-0.602397	-0.505905
44	6	0	-5.919133	-2.916289	-1.923545
45	1	0	-3.904127	-2.369710	-1.532472
46	6	0	-7.661697	-1.424292	-1.038444
47	1	0	-7.008791	0.326175	-0.041465
48	6	0	-7.280502	-2.590539	-1.738770
49	1	0	-5.653990	-3.829697	-2.442959
50	1	0	-8.712280	-1.172401	-0.948298
51	1	0	-8.047324	-3.243354	-2.146021
52	6	0	-4.591301	0.911897	0.909184
53	6	0	-4.199599	2.246393	0.896590
54	6	0	-5.779621	1.299247	2.955586
55	1	0	-5.635006	-0.487873	2.009231
56	6	0	-4.642912	3.122059	1.897989
57	1	0	-3.601866	2.619512	0.075598
58	6	0	-5.438686	2.648679	2.936167
59	1	0	-6.371259	0.844071	3.742204
60	1	0	-4.371966	4.171517	1.856128
61	1	0	-5.794622	3.299969	3.726240
62	7	0	-1.850170	0.009990	-0.010874
63	7	0	1.805191	0.020714	-0.061769
64	7	0	6.559687	-0.576839	0.895943
65	7	0	-5.369646	0.493821	1.959362

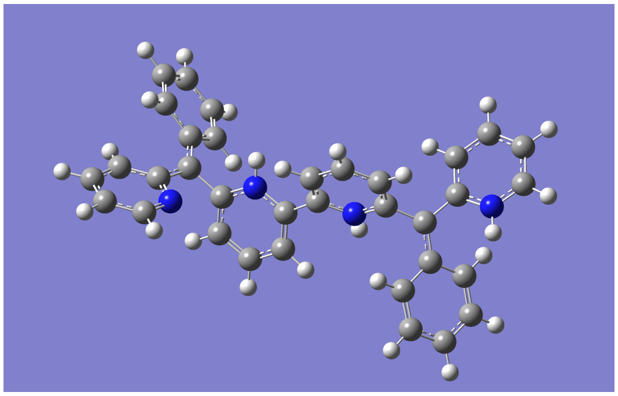

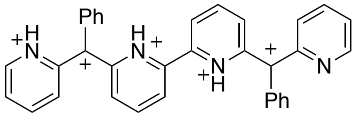

21

Imaginary Frequencies=0

```
Zero-point correction=                           0.527198 (Hartree/Particle)
 Thermal correction to Energy=                    0.557544
 Thermal correction to Enthalpy=                  0.558488
 Thermal correction to Gibbs Free Energy=         0.462062
 Sum of electronic and zero-point Energies=          -1528.640854
 Sum of electronic and thermal Energies=             -1528.610507
 Sum of electronic and thermal Enthalpies=           -1528.609563
 Sum of electronic and thermal Free Energies=        -1528.705989
```

Standard orientation:

Center Number	Atomic Number	Atomic Type	Coordinates (Angstroms) X	Y	Z
1	6	0	-1.736709	-1.998465	-1.981681
2	6	0	-0.587244	-1.600427	-1.278075
3	6	0	-0.680141	-0.643550	-0.283982
4	6	0	-3.061754	-0.492993	-0.645189
5	6	0	-2.971183	-1.465859	-1.649681
6	1	0	-1.660508	-2.751328	-2.759436
7	1	0	0.353933	-2.093168	-1.487902
8	1	0	-2.010511	0.637761	0.664770
9	1	0	-3.873740	-1.793412	-2.151962
10	6	0	0.458460	-0.261554	0.597321
11	6	0	0.299038	0.010600	1.958874
12	6	0	2.851197	0.073016	0.828713
13	1	0	1.851526	-0.384914	-0.909712
14	6	0	1.401463	0.335677	2.743231
15	1	0	-0.678679	-0.071823	2.418830
16	6	0	2.679360	0.382867	2.172579
17	1	0	1.275060	0.534513	3.803314
18	1	0	3.537496	0.617983	2.790065

19	6	0	4.211166	0.036125	0.194344
20	6	0	-4.388386	0.119374	-0.241142
21	6	0	-4.770315	1.394317	-0.703830
22	6	0	-5.818665	2.135168	-0.049624
23	6	0	-4.175207	1.965282	-1.883450
24	6	0	-6.283633	3.314796	-0.584912
25	1	0	-6.196539	1.798138	0.906810
26	6	0	-4.669155	3.129889	-2.419576
27	1	0	-3.393081	1.432395	-2.412317
28	6	0	-5.730059	3.806333	-1.780358
29	1	0	-7.060637	3.876198	-0.079229
30	1	0	-4.262634	3.526623	-3.342084
31	1	0	-6.109622	4.729242	-2.207363
32	6	0	4.660443	-1.069796	-0.543957
33	6	0	4.080814	-2.387798	-0.355973
34	6	0	5.768192	-0.946832	-1.478216
35	6	0	4.614344	-3.480442	-0.989097
36	1	0	3.302064	-2.534309	0.383278
37	6	0	6.256720	-2.045736	-2.138481
38	1	0	6.155642	0.034115	-1.727257
39	6	0	5.701329	-3.321544	-1.885340
40	1	0	4.232976	-4.475550	-0.788476
41	1	0	7.054827	-1.942149	-2.866042
42	1	0	6.111511	-4.193574	-2.388636
43	6	0	5.081667	1.200041	0.468761
44	6	0	4.645841	2.523628	0.453320
45	6	0	5.551207	3.577681	0.662130
46	1	0	3.609810	2.748695	0.234298
47	6	0	7.298878	1.966063	0.980210
48	1	0	6.756043	0.026075	0.824202
49	6	0	6.886974	3.301460	0.927431
50	1	0	5.204618	4.605261	0.617758
51	1	0	8.314017	1.667242	1.223376
52	1	0	7.610568	4.089681	1.109131
53	6	0	-5.156038	-0.786835	0.567615
54	6	0	-6.569153	-0.772282	0.667221
55	6	0	-4.991762	-2.623361	1.951303
56	6	0	-7.189389	-1.715020	1.481950
57	1	0	-7.166139	-0.090404	0.076121
58	6	0	-6.395997	-2.644576	2.145500
59	1	0	-4.368555	-3.365521	2.439348
60	1	0	-8.269343	-1.739813	1.573314
61	1	0	-6.838331	-3.404689	2.780347
62	7	0	-4.386210	-1.739564	1.180659
63	7	0	-1.919211	-0.096120	-0.034148
64	7	0	1.729340	-0.229467	0.089436
65	7	0	6.416712	0.983043	0.739456
